# The Role of Toll-Like Receptor 2 in Inflammation and Fibrosis during Progressive Renal Injury

**DOI:** 10.1371/journal.pone.0005704

**Published:** 2009-05-27

**Authors:** Jaklien C. Leemans, Loes M. Butter, Wilco P. C. Pulskens, Gwendoline J. D. Teske, Nike Claessen, Tom van der Poll, Sandrine Florquin

**Affiliations:** 1 Department of Pathology, Academic Medical Center, University of Amsterdam, Amsterdam, the Netherlands; 2 Center for Experimental and Molecular Medicine, Academic Medical Center, University of Amsterdam, Amsterdam, the Netherlands; Institut Pasteur, France

## Abstract

Tissue fibrosis and chronic inflammation are common causes of progressive organ damage, including progressive renal disease, leading to loss of physiological functions. Recently, it was shown that Toll-like receptor 2 (TLR2) is expressed in the kidney and activated by endogenous danger signals. The expression and function of TLR2 during renal fibrosis and chronic inflammation has however not yet been elucidated. Therefore, we studied TLR2 expression in human and murine progressive renal diseases and explored its role by inducing obstructive nephropathy in TLR2^−/−^ or TLR2^+/+^ mice. We found that TLR2 is markedly upregulated on tubular and tubulointerstitial cells in patients with chronic renal injury. In mice with obstructive nephropathy, renal injury was associated with a marked upregulation and change in distribution of TLR2 and upregulation of murine TLR2 danger ligands Gp96, biglycan, and HMGB1. Notably, TLR2 enhanced inflammation as reflected by a significantly reduced influx of neutrophils and production of chemokines and TGF-β in kidneys of TLR2^−/−^ mice compared with TLR2^+/+^ animals. Although, the obstructed kidneys of TLR2^−/−^ mice had less interstitial myofibroblasts in the later phase of obstructive nephropathy, tubular injury and renal matrix accumulation was similar in both mouse strains. Together, these data demonstrate that TLR2 can initiate renal inflammation during progressive renal injury and that the absence of TLR2 does not affect the development of chronic renal injury and fibrosis.

## Introduction

Tissue fibrosis is a hallmark of a variety of chronically failing organs, including progressive renal disease, and is a leading cause of morbidity and mortality worldwide [1]. A growing body of evidence suggest that almost 45% of all deaths in the developed world can be ascribed to some type of chronic fibrotic disease such as interstitial pulmonary fibrosis, liver cirrhosis, and progressive renal disease [1]. In the kidney, the degree of inflammation and fibrosis of the tubulointerstitial compartment are strong predictive factors for the loss of renal function and the risk for progression to end-stage renal disease [2]. Renal fibrosis is defined by the accumulation of interstitial leukocytes and myofibroblasts that contribute to abnormal accumulation of extracellular matrix (ECM) and eventual tubular atrophy and loss of renal function [3]–[5]. Irrespective of the nature of the primary disease and the originating renal compartment, renal fibrosis is considered to be the common final pathway by which kidney diseases with variable etiology progresses to end-stage renal failure. It is therefore important to identify factors that participate in the initiation of tubulointerstitial inflammation and subsequent interstitial fibrosis during progressive renal injury. From our own and other data, it has become clear that Toll-like receptor (TLR)2 and 4 plays a crucial role in the induction of acute inflammation and early tubular injury in the kidney in a reversible model of acute renal injury [6]–[8].

TLR2 belongs to the TLR family of conserved pattern recognition receptors that are expressed on leukocytes, (myo)fibroblasts [9], [10], and renal cells [11], [12] and can detect motifs of pathogens and subsequently induce innate and adaptive immunity. Most interestingly, TLRs, including TLR2, also recognize endogenous danger molecules that have been altered from their native state or accumulate in non-physiologic sites or amounts during cellular injury [13]–[22]. In this context, TLRs may be crucial cellular sentinels to detect “danger” signals released during tissue damage [23] and important initiators of inflammation and fibrosis. The expression and localization of TLRs has however not been analyzed previously during fibrinogenesis, and nothing is known about their role in renal progressive disease and fibrosis. A potential role for TLRs in the physiopathological pathway of fibrosis is suggested by a recent observation showing that TLR4 enhances myofibroblast activation and fibrinogenesis in the liver [24]. Together, these data led to the hypothesis that TLR2 plays a key role in monitoring renal injury and in mediating inflammation and fibrosis in progressive renal disease. To test this hypothesis we studied the expression of TLR2 in human and murine chronic renal diseases and explored its role by inducing renal fibrosis in TLR2^−/−^ or TLR2^+/+^ mice using the murine model of unilateral ureteral obstruction (UUO), a well accepted experimental model for the study of the mechanisms of progressive renal fibrosis.

## Results

### TLR2 is upregulated in kidneys from patients with tubulo-interstitial damage

**Figure 1:**
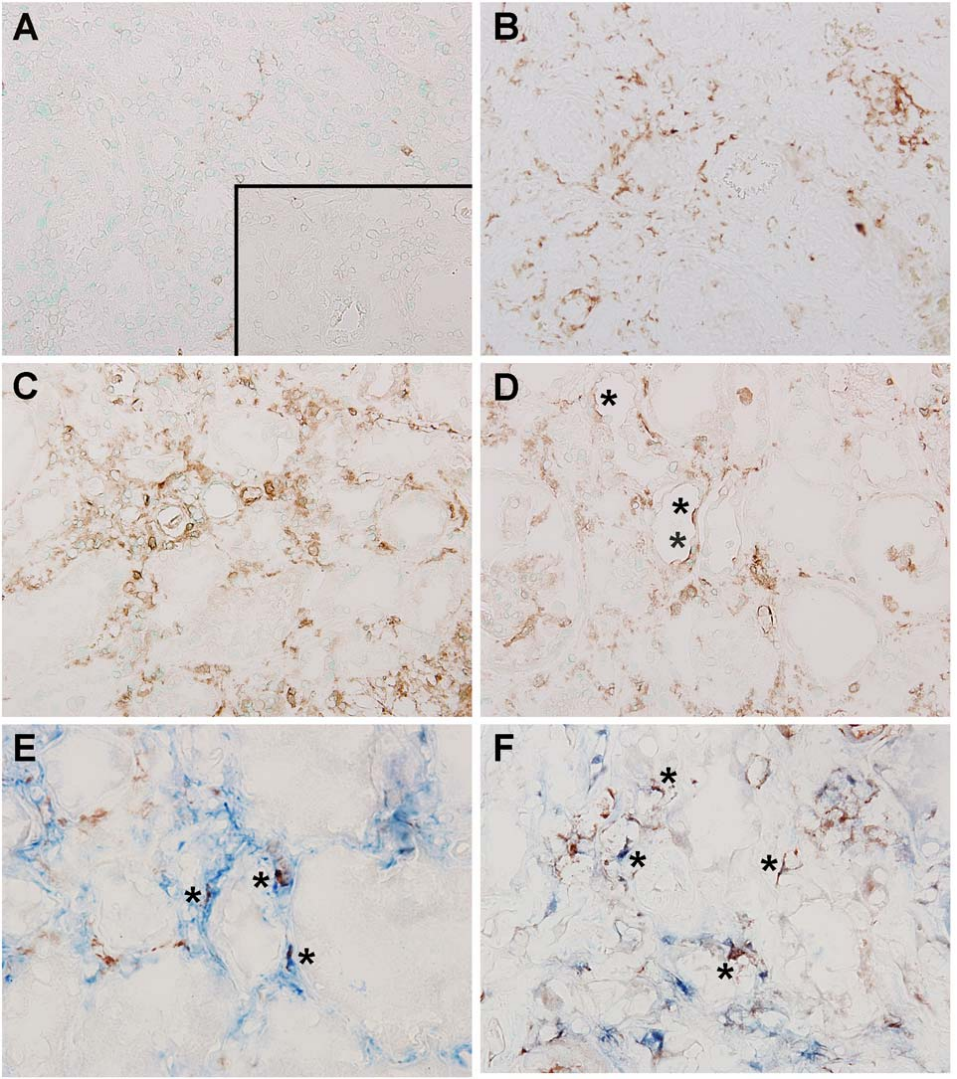
TLR2 expression and localization in renal biopsy specimens of patients with obstructive hydronephrosis (B), severe forms of IgA nephropathy (C, D) or in control renal biopsy specimens (A). TLR2 was clearly upregulated in kidneys of patients with obstructive hydronephrosis or IgA nephropathy and mainly expressed by tubulointerstitial cells. Some expression was found at the apical side of renal tubules (asterisks, D). Double staining showed coexpression of various myofibroblasts (blue) (asterisks E), and numerous macrophages (blue) with TLR2 (red) (asterisks F). No immunoreactivity of TLR2 was observed in negative control sections derived from IgA nephropathy patients (insert A, primary antibody omitted).

Fibrosis and renal atrophy are common outcomes of hydronephrosis due to obstruction. Therefore, we wondered if tubulo-interstitial damage that developed upon obstructive hydronephrosis may be associated with TLR2 expression. Immunohistochemical staining for TLR2 with an Ab that was proven to be highly specific for both murine and human TLR2 [6], [25] revealed a strong increase of TLR2 expression in every one of the four analyzed hydronephrotic obstructed kidneys of patients (Fig 1B) compared to control biopsies without tubular changes and with few TLR2 positive interstitial cells (Fig. 1A). Extensive renal atrophy in end-stage hydronephrotic kidneys made it however impossible to phenotype TLR2-positive cells on histologic sections. Therefore, we analyzed TLR2 expression during chronic IgA nephropathy which is also associated with interstitial fibrosis, tubular atrophy, and interstitial inflammation. Similar to patients with hydronephrosis we found a strong increase of TLR2 expression in all the fifteen analyzed kidneys of IgA nephropathy patients (Fig. 1C and D) compared to control renal biopsy specimens (Fig. 1A). TLR2 was mainly expressed by tubulointerstitial cells and some expression was found at the apical side of renal tubules (asterisks Fig. 1D). Double staining revealed that various interstitial myofibroblasts (blue) (asterisks Fig. 1E) and in particular macrophages (blue) (asterisks Fig. 1F) expressed TLR2 (red). Occasionally, we found granulocytes in renal biopsies with low expression of TLR2 (data not shown).

### TLR2 and its endogenous stress ligands are markedly upregulated in kidneys during UUO in mice

**Figure 2:**
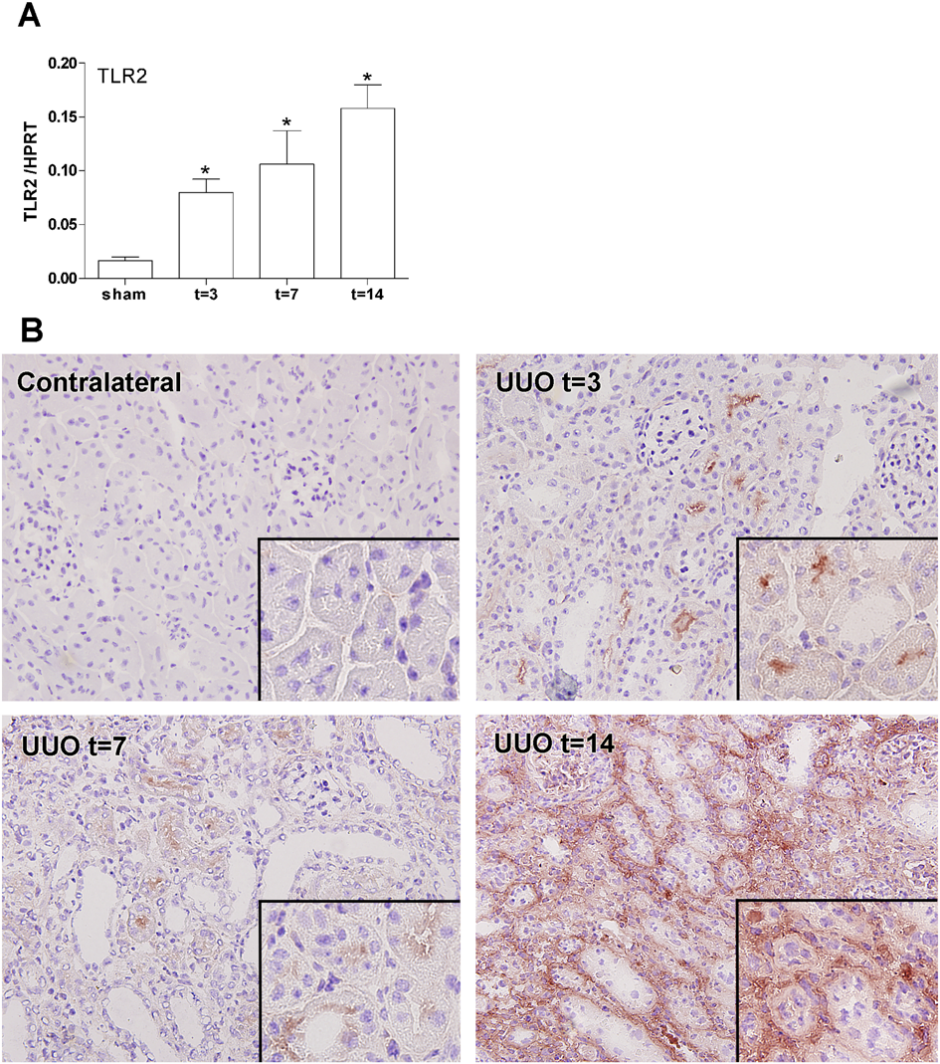
Expression and localization of TLR2 in UUO-injured kidney of TLR2^+/+^ mice at several time points. The amount of TLR2 mRNA (A) and protein (B) was markedly upregulated after UUO injury. Data are mean and SEM of six mice per group; *p<0.05. High magnification images (inserts) show that 3 and 7 days after UUO TLR2 was mainly located at the apical side of renal tubules in both cortex and medulla. After 14 days, apical tubular TLR2 staining in cortex and medulla is lost and extended into the cytoplasm and the interstitium.

**Figure 3:**
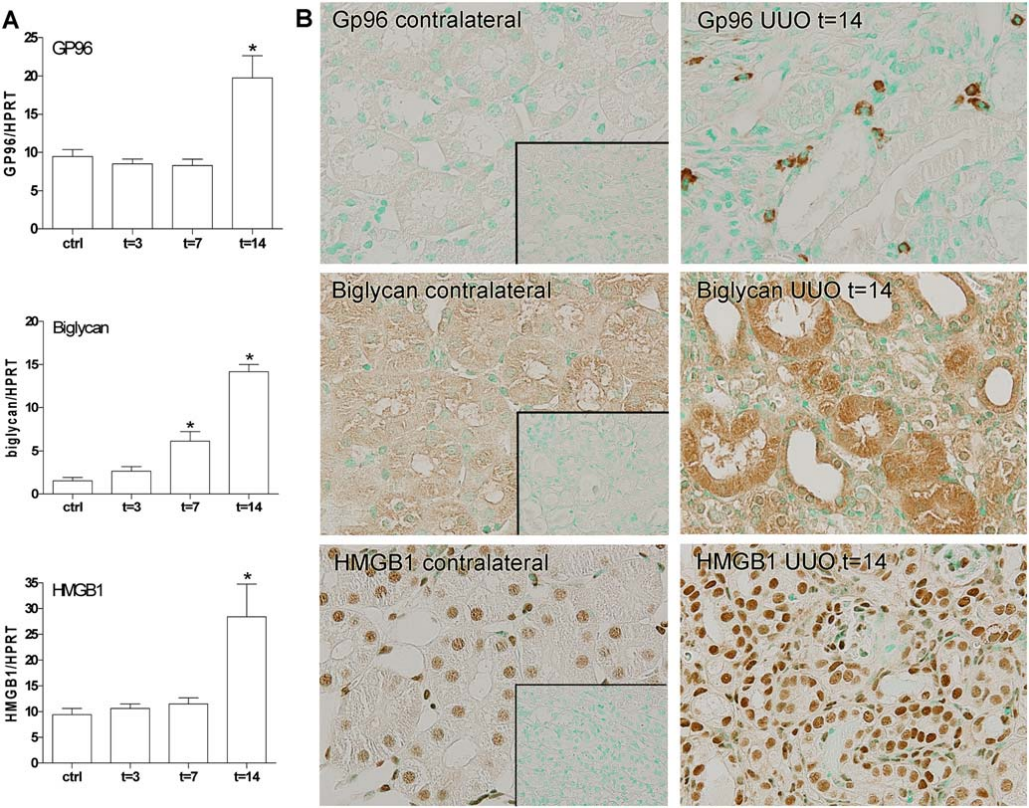
Expression and localization of endogenous stress ligands in UUO-injured kidney of TLR2^+/+^ mice at several time points. The amount of biglycan, HMGB1, and Gp96 mRNA (A) and protein(B) was markedly upregulated after UUO injury. Data are mean and SEM of six mice per group; *p<0.05. Inserts show the negative controls (t = 14) omitting primary antibody.

To evaluate the influence of fibrinogenesis at different stages on the expression and localization of TLR2 we next measured the amount of mRNA and protein in UUO-injured kidney of TLR2^+/+^ mice at several time points. This revealed that the amount of TLR2 mRNA was almost 5 times increased 3 days after UUO and continued to increase at later time points (Fig. 2A). The expression pattern of TLR2 protein followed a similar trend as compared to TLR2 mRNA expression. TLR2 protein was markedly upregulated 3, 7 and 14 after UUO-injury in kidneys of TLR2^+/+^ mice as compared with the contralateral unobstructed kidneys (Fig. 2B). High magnification images (inserts) show that 3 and 7 days after the induction of injury TLR2 was predominantly located at the apical side of renal tubules. After 14 days, apical TLR2 staining seems to be lost and in stead extended into the cytoplasm and interstitium. Control sections (t = 14), omitting primary antibody showed no overt staining (data not shown). Analysis of the expression of known endogenous stress ligands that potentially could activate TLR2, e.g. Gp96, biglycan, HMGB1, HSP60 and HSP70 [16], [19], [26]–[28] during progressive renal injury revealed a strong upregulation of the amount of biglycan (t = 7, 14), HMGB1 (t = 14), and Gp96 (t = 14) mRNA after UUO injury compared to contralateral kidneys (Fig. 3A). In line, we found a profound upregulation of tubulointerstitial Gp96, tubular biglycan and HMGB1 protein 14 days after the induction of UUO injury (Figure 3B). No upregulation was seen in HSP70 and HSP60 mRNA levels after UUO-injury (data not shown). Together, these data suggest that progressive renal injury leads to a marked enhancement of TLR2 and several main endogenous stress ligands.

### No effect of TLR2 deficiency on UUO-induced tubular injury and ECM accumulation

**Figure 4:**
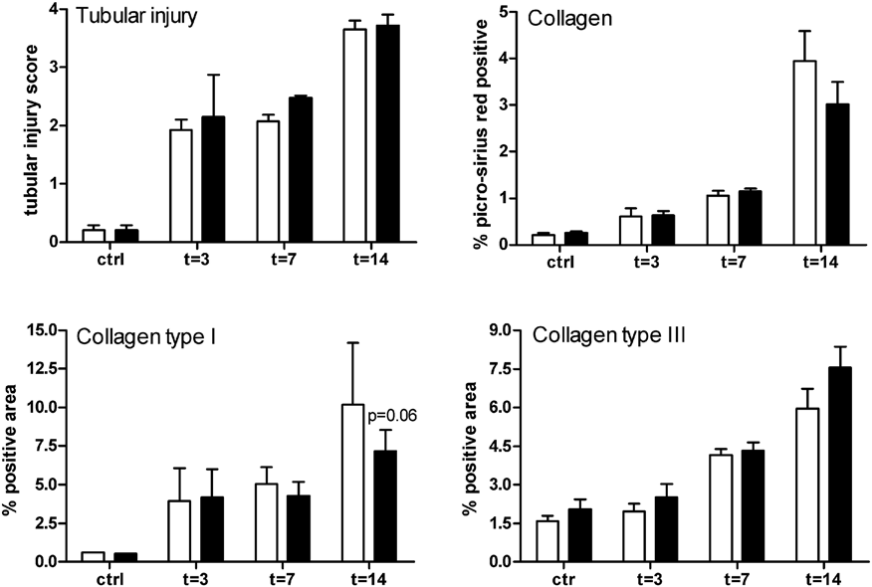
Analysis of UUO-induced tubular injury and fibrosis. Tubular injury was semi-quantitatively scored using PAS-D stained renal tissue sections. Tubular damage was similar between TLR2^+/+^ (□) and TLR2^−/−^ (▪) mice at all time points. Fibrosis was quantitatively scored by measuring renal collagen accumulation in TLR2^+/+^ and TLR2^−/−^ mice 3, 7, and 14 days after UUO or in contralateral kidneys. This revealed that fibrosis was comparable in kidney of TLR2^−/−^ mice compared with TLR2^+/+^ animals during UUO-induced injury. The percentage of positive staining for collagen (picrosirius red staining), collagen type I and III was analyzed in both cortex and medulla using a computer-assisted digital analysis program. Data are mean and SEM of six mice per group, *p<0.05.

To assess the functional contribution of TLR2 to the development of tubular injury and interstitial fibrosis in UUO, we next scored for histological signs of renal injury and analyzed the expression of the major ECM component collagen. This revealed that tubular injury increased markedly after UUO as reflected by the presence of dilated tubules, loss of brush border, and epithelial simplification in the tubulointerstitium (data not shown). However, semi-quantitative scoring of these renal sections revealed no differences in renal injury between both groups of animals (Fig 4). Fibrosis increased progressively from day 3 till 14 as reflected by a marked upregulation of collagen in obstructed kidneys of both animal strains compared to the contralateral kidney (Fig. 4). However, no differences in collagen accumulation could be found between both mouse strains as assessed by picrosirius red staining and collagen type I and III immunostaining (Fig 4).

### Reduced renal inflammation in TLR2^−/−^ mice

**Figure 5:**
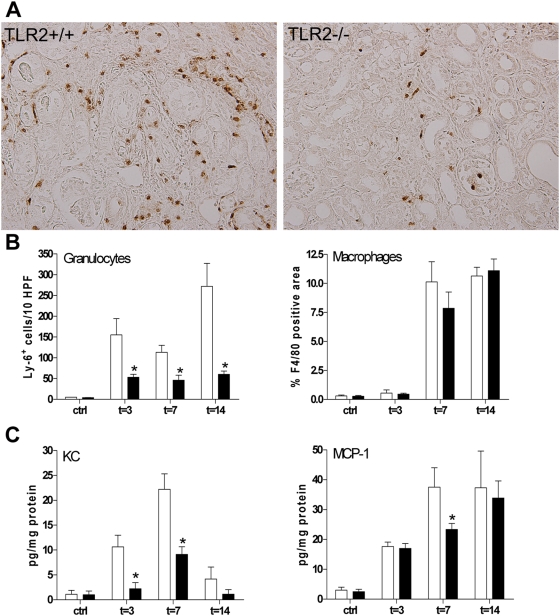
Leukocyte influx (A, B) and chemokine (C) levels in kidneys from TLR2^+/+^ (□) and TLR2^−/−^ (▪) mice 3, 7, and 14 days after UUO or in contralateral kidneys. The number of granulocytes (B, t = 3, 7, 14) were significantly lower in TLR2^−/−^ mice compared to TLR2^+/+^ animals after UUO as counted in 10 randomly selected high-power fields (A, t = 14). No differences were found in the amount of macrophages. Leukocytes were counted in cortex and medulla of renal tissue sections stained for Ly-6G (A: t = 14, B) and F4/80 (B). TLR2^−/−^ mice have furthermore significantly reduced KC (t = 3, 7, 14), and MCP-1 (t = 7) levels in their kidneys as compared to kidneys from TLR2+/+ mice (C). The presence of protein was measured in kidney homogenates by specific ELISA. Data are mean and SEM of six mice per group. *p<0.05.

One of the early events in progressive renal injury is the recruitment of inflammatory cells. Analysis of the influx of granulocytes and macrophages demonstrated that the amount of inflammatory cells increased progressively from day 3 till 14 in obstructed kidneys compared to the contralateral unobstructed kidney (Fig. 5B). The increased leukocyte influx in the tubulointerstitium was accompanied by a strong increase in renal KC (granulocyte attractant), and MCP-1 (monocyte chemoattractant) (Fig. 5C). Interestingly, granulocyte influx was 3, 2.5 and 4.5 times lower in the obstructed kidney of TLR2^−/−^ animals compared with TLR2^+/+^ mice respectively 3, 7, and 14 days after UUO injury (Fig. 5A,B) as assessed by immunohistochemistry (Fig. 5A, t = 14). In line, we found that the levels of KC (t = 3, t = 7) was significantly lower in the obstructed kidney of TLR2^−/−^ animals compared with TLR2^+/+^ mice (Fig. 5C). Although no significant differences were found when analyzing macrophage influx (Fig. 5B) we did find lower levels of MCP-1 (t = 7) in TLR2^−/−^ mice compared to TLR2^+/+^ animals. Together, these results reveal an important role for TLR2 in the induction of a pro-inflammatory immune response during progressive renal injury.

### Reduced amounts of myofibroblasts in TLR2^−/−^ mice

**Figure 6:**
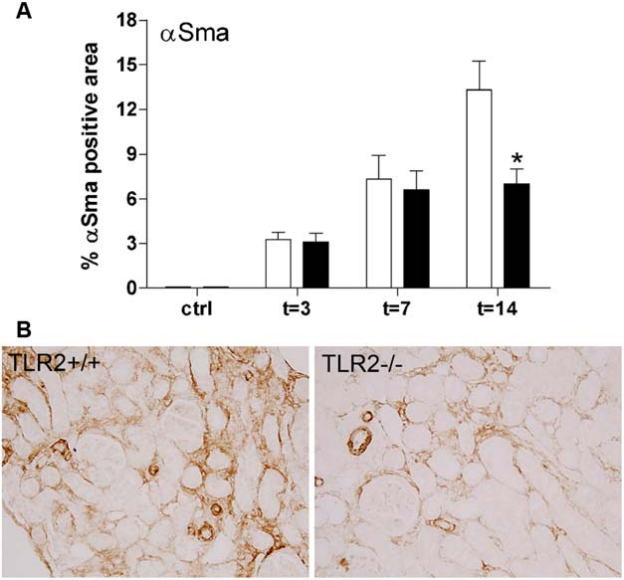
Quantitative analysis of the amount of interstitial myofibroblasts (α-SMA) in TLR2^+/+^ (□) and TLR2^−/−^ (▪) mice 3, 7, and 14 days after UUO or in contralateral non-obstructed kidneys as a cellular marker of fibrosis (A). This revealed that the amount of α-SMA was increased during UUO and was significantly lower in kidney of TLR2^−/−^ mice compared with TLR2^+/+^ animals 14 days after UUO-induced injury. The percentage of positive staining for myofibroblasts was analyzed in both cortex and medulla using a computer-assisted digital analysis program on renal tissue sections stained for α-SMA (B: t = 14). Data are mean and SEM of six mice per group, *p<0.05.

To further explore the role of TLR2 during obstructive nephropathy, we additionally analyzed the amount of myofibroblasts (α-SMA). This showed that the amount of myofibroblasts was significantly increased 3 days post-UUO and continued to increase at later time points. Interestingly, significantly less myofibroblasts accumulated in the renal peritubular interstitium of TLR2^−/−^ mice compared with TLR2^+/+^ animals 14 days after UUO-injury (Fig. 6).

### Reduced activation of Matrix Metalloproteinases in TLR2^−/−^ mice

**Figure 7:**
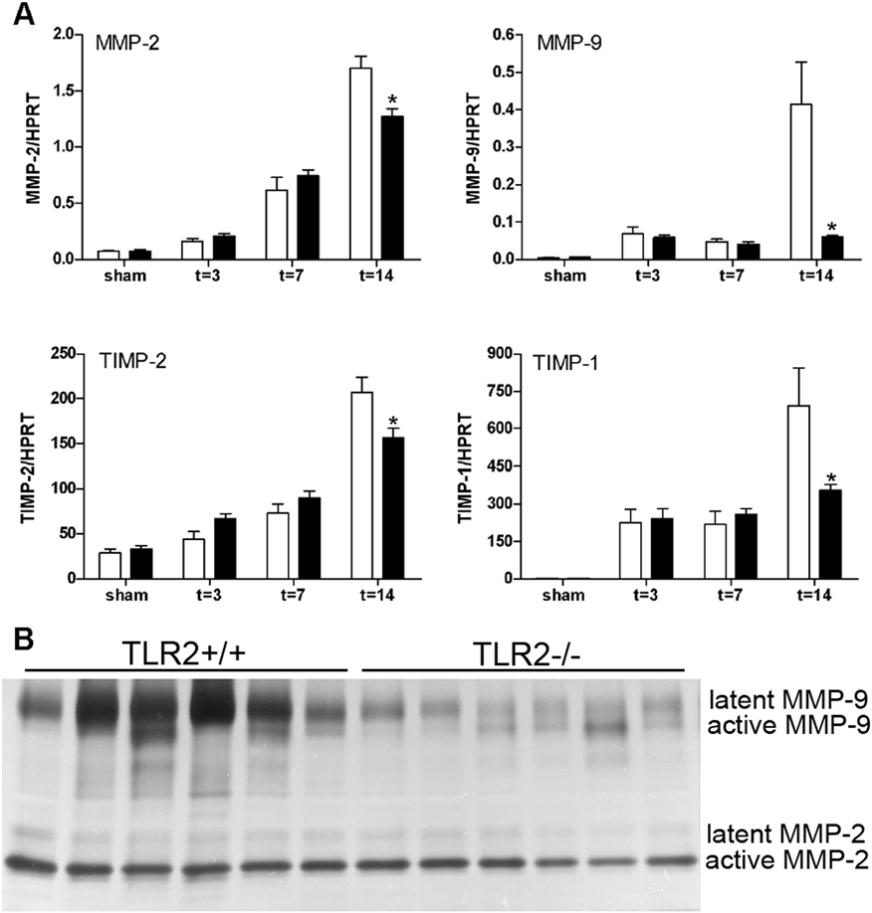
Expression (A) and activity (B) of the MMP-mediated collagen degradation pathway in TLR2^+/+^ (□) and TLR2^−/−^ (▪) mice 3, 7, and 14 days after UUO or in contralateral non-obstructed kidneys. Both MMPs and TIMPs were significantly reduced in TLR2^−/−^ mice compared to TLR2^+/+^ mice 14 days after UUO-injury (A). Data are mean and SEM of six mice per group, *p<0.05. Gel zymography (B) illustrates that renal MMP-9 activity was lower in kidneys TLR2^−/−^ mice at this time point. No difference between TLR2^−/−^ and TLR2^+/+^ mice was observed for MMP-2 activity at 14 days post-UUO.

We next investigated the main pathway that is involved in ECM degradation: the matrix metalloproteinases (MMP) degrading pathway. This demonstrated that progressive renal injury increased mRNA expression of MMP2 and MMP9 and their respective inhibitors TIMP2 (tissue inhibitors of metalloproteinases) and TIMP1 (Fig. 7A). Interestingly, both MMPs and TIMPs were significantly reduced in TLR2^−/−^ mice compared to TLR2^+/+^ mice 14 days after UUO-injury (Fig. 7A). The decrease in mRNA of MMP2 and MMP9 was accompanied with a lower MMP9 activity in kidneys TLR2^−/−^ mice as assessed by zymography at this time point (Fig. 7B, no difference after 7 days, data not shown). No difference between TLR2^−/−^ and TLR2^+/+^ mice was observed for MMP-2 activity both 7 and 14 days post-UUO.

### TLR2^−/−^ mice have lower levels of apoptosis in UUO-induced fibrosis

**Figure 8:**
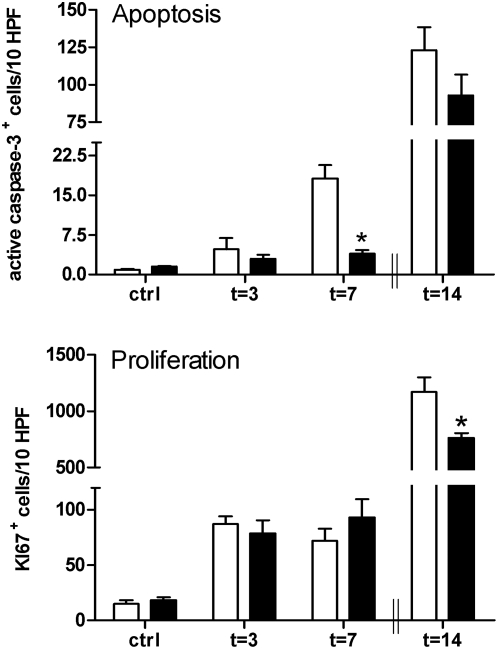
Apoptotic and proliferating tubular cells in kidneys from TLR2^+/+^ (□) and TLR2^−/−^ (▪) mice 3, 7, and 14 days after UUO or in contralateral kidneys. The number of apoptotic (t = 7) tubular cells and proliferating (t = 14) cells were significantly lower in kidneys from TLR2^−/−^ mice compared to kidneys from TLR2^+/+^ animals as counted in 10 randomly selected high-power fields. Cells were counted on renal tissue sections stained for active caspase-3 (apoptosis) and Ki67 (proliferation). Due to severe tubular atrophy, it is impossible to identify apoptotic TECs after 14 days. Therefore we analyzed at this time point the total amount of apoptotic cells (both interstitial cells and tubular cells). Data are mean and SEM of six mice per group, *p<0.05.

Tubulointerstitial injury in UUO-injury can result in an imbalance between tubular epithelial cell (TEC) apoptosis and proliferation. We found that apoptosis occurred predominantly in the renal cortex and increased significantly after 3 days of obstruction. A marked decrease in the number of apoptotic TECs was present in obstructed kidneys of TLR2^−/−^ mice compared to TLR2^+/+^ kidneys at day 7 (Fig. 8A). As it is difficult, if not impossible, to identify apoptotic TECs after 14 days when tubular atrophy was very severe we analyzed at this time point the total amount of apoptotic cells and found a similar trend as seen after 7 days. Tubular proliferation of cortical tubules became prominent on day 3 post-UUO (Fig. 8B). However, no differences were found between TLR2^−/−^ and TLR2^+/+^ at 3 and 7 days after obstruction. Fourteen days after obstruction, TLR2^−/−^ mice showed significantly less proliferation of cells than TLR2^+/+^ animals.

### Reduced Renal TGFβ activity

**Figure 9:**
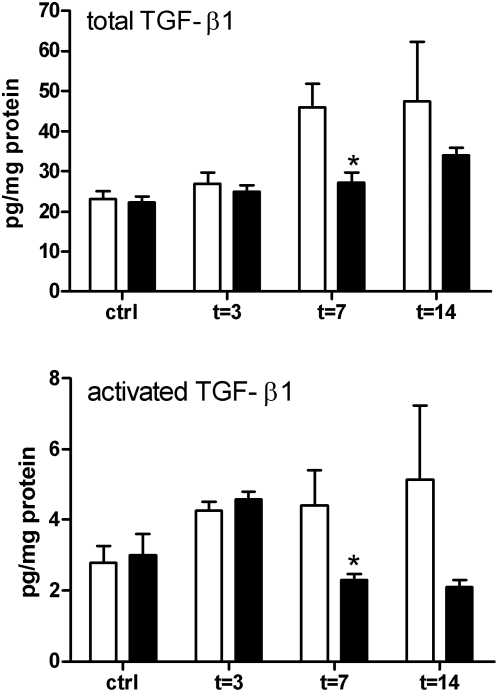
Total and active TGF-β levels in kidneys from TLR2^+/+^ (□) and TLR2^−/−^ (▪) mice 3, 7, and 14 days after UUO or in contralateral non-obstructed kidneys. The amount of total TGFβ and activated TGFβ was significantly lower in kidneys from TLR2^−/−^ mice compared to TLR2^−/−^ mice 7 days after UUO-induced injury. Data are mean and SEM of six mice per group. *p<0.05.

TGFβ is one of the most important molecules in the pathogenesis of renal fibrogenesis [29]. To determine whether the reduction in renal fibrosis and apoptosis in TLR2^−/−^ mice was associated with an alteration in TGFβ, we finally examined TGFβ protein. This revealed that the amount of total TGFβ as well as activated TGFβ was significantly lower in kidneys from TLR2^−/−^ mice compared to TLR2^+/+^ mice 7 days after UUO-induced injury (Fig. 9). A trend towards less (active) TGFβ was furthermore seen 14 days after UUO-induced injury (not significant). As HGF antagonizes the fibrogenic effects of TGFβ, we also analyzed the renal level of this growth factor and found no significant differences between TLR2^+/+^ and TLR2^−/−^ mice at all time points (data not shown).

## Discussion

Renal fibrosis is the final common pathway for numerous kidney diseases regardless of the initiating pathology that can cause progressive renal failure. Therefore, knowledge of the regulatory mechanisms involved in monitoring a primary renal insult, and in mediating renal fibrosis and infammation is essential for the development of therapeutic strategies for the prevention or treatment of progressive kidney diseases. Although we have previously shown that TLR2 plays a critical role in the initiation of acute renal inflammation and early tubular injury in a reversible model of acute renal ischemic injury [6], nothing was known about its role in chronic inflammation and fibrosis during progressive renal injury. Our results demonstrate that TLR2 and its main stress ligands are markedly upregulated in the kidney after UUO-injury, obstructive hydronephrosis (TLR2) and IgA nephropathy (TLR2) and identify TLR2 as an initiator of renal inflammation (neutrophil influx, cytokine induction) during progressive renal injury. TLR2 does not play an essential role in the development of renal fibrosis or the progression of renal disease after UUO-injury.

In this study we characterized the expression pattern of TLR2 in kidneys from patients with obstructive hydronephrosis, severe IgA nephropathy as well as from mice subjected to obstructive nephropathy. We found a clear increase in TLR2 protein expression in patients with progressive renal injury as well as an increase in TLR2 mRNA and protein through a time course of UUO with a prominent change in the distribution pattern. During early phases of UUO TLR2 was mainly located at the apical site of tubules while it was lost and in stead extended throughout the tubular cytoplasm and the interstitium at later time points when fibrosis was more severe. In agreement, we found that patients with severe IgA nephropathy had a predominant interstitial TLR2 expression with occasionally apical staining of tubuli. The apical expression of TLR2 on tubular cells could be optimal for interaction with filtered or leaked stress ligands [30]–[32] or with ligands that are expressed apically [33]. Indeed, we found a profound upregulation of tubular biglycan with expression at the apical side and in the cytoplasm during UUO injury. Moreover, there are data demonstrating that HMGB1 can be secreted from cells and can leak into the urine [34] making it possible to interact with apical TLR2. Although cytoplasmic HMGB1 was only faintly found, it could as well interact with cytoplasmic TLR2. Finally, tubulointerstitial cell-associated Gp96 might interact with TLR2-positive tubulointerstitial cells at the later phases of UUO. One has to keep in mind that these ligands could potentially activate TLR4 as well. The intracellular localization of TLR2 is consistent with data demonstrating that upon stimulation by specific ligands, TLR2 receptor clusters are targeted to the Golgi apparatus [35]. Loss of apical TLR2 staining in tubuli in later phases of UUO or during severe forms of IgA nephropathy could reflect loss of apical brush border due to tubular damage. Of particular interest was the observation that several renal myofibroblasts coexpressed TLR2. In line, others have found that intestinal myofibroblasts and synovial fibroblasts of patients with rheumatoid arthritis express TLR2 [9], [10]. TLR2 positive interstitial cells in IgA nephropathy patients and mice subjected to UUO therefore most likely reflect the influx of leukocytes known to express TLRs and the accumulation of myofibroblasts. Indeed, we found numerous TLR2-positive macrophages in patients with IgA nephropathy. Although Wolfs *et al.* found constitutive TLR2 mRNA expression in murine tubular epithelial cells of normal kidney [12], we could only detect TLR2 protein in tubular cells when the kidney was damaged. This might indicate that the antibody we used is not sensitive enough to detect very low levels of protein or that some sort of regulation of the TLR2 expression is being applied at the post-transcriptional, translational or proteosomal level.

With the use of TLR2^−/−^ mice, we additionally demonstrated that TLR2 did not play an essential role in the development of progressive renal injury and fibrosis during obstructive nephropathy. It is generally believed that fibrosis of several organs, including the kidney, is a consequence of the disturbance of the balance between ECM synthesis by myofibroblasts and ECM degradation by matrix metalloproteinases as well as by excessive and persistent inflammation. Although we found significantly less myofibroblasts in TLR2^−/−^ mice compared to Wt animals 14 days post-UUO, the accumulation of ECM components was not different between both mouse strains. The latter is in agreement with findings of Seki *et al.* which show that TLR2 is not required for hepatic fibrogenesis after bile duct ligation [24]. The decrease in myofibroblasts can be explained by the reduction in fibroblasts activation. Fibroblasts become activated into myofibroblasts by stimuli such as the infiltration of inflammatory cells and TGF-β1 [36]. Indeed, we found that both the amount of granulocytes as well as the amount of TGF-β1 was significantly decreased in mice deficient for TLR2 compared to Wt animals. In line with the similar degree of fibrosis and injury, no differences were found in the amount of infiltrated macrophages in TLR2^+/+^ and TLR2^−/−^ mice.

Next, we investigated analyzed the MMP degrading pathway. Surprisingly, MMP2/TIMP2 and MMP9/TIMP1 mRNA expression and MMP9 activity was considerably reduced in TLR2^−/−^ mice compared to Wt animals. This is in line with findings demonstrating that MMP9 is selectively induced through TLR2 in human and murine monocytic cells stimulated with bacterial TLR ligands [37], [38]. The diminished MMP activity in TLR2^−/−^ mice would explain why there is no difference in ECM accumulation in these animals despite the reduction of matrix-producing myofibroblasts and TGF-β. Indeed, it is generally assumed that diminished MMP activity is responsible for ECM deposition.

In addition, we investigated the role of TLR2 in inflammation during obstructive nephropathy. The observations that renal TECs, macrophages and myofibroblasts express TLR2 [9]–[12], [39](this study) and that the main TLR2 danger ligands are upregulated during progressive renal injury (this study) suggest that these cells are capable of monitoring cellular damage and inducing an inflammatory response. Indeed, we found that TLR2^−/−^ mice had reduced chemokine levels and neutrophil influx in their kidneys compared to Wt animals during obstructive nephropathy. Although the presence and kinetics of neutrophils are not well documented during UUO and neutrophils are not a major player in this model, Duymelinck *et al.* as well demonstrate the presence of PMNs in UUO-kidneys [40]. The significance of this finding is however not clear and further studies will be necessary to elucidate the exact role of neutrophils in this model. We and others have shown that TLR2 and TLR4 is needed for chemokine production of murine TECs that are exposed to simulated ischemia in vitro and that renal-associated TLR is an important initiator of inflammatory responses in ischemic acute renal failure in vivo [6]–[8]. In addition, others have shown that murine TECs, macrophages and human (myo)fibroblasts produce chemokines via TLRs when exposed to bacterial components [9], [11], [41] or endogenous danger ligands [42]. A further explanation for the decrease in cytokines and TGF-β in mice lacking TLR2 could be that there is a reduction in TLR2-dependent macrophage and (myo)fibroblast activation. This would agree with our observations that various renal myofibroblasts and in particular macrophages express TLR2. Indeed, TLR ligands provide a major activating signal to macrophages [42] and (myo)fibroblasts [9], [41], [43] via TLRs which on their turn can produce cytokines among which TGF-β. In line with this thought is a recent paper demonstrating that TLR4 induce myofibroblast activation, TGF-β signaling, chemokine production and fibrinogenesis in the liver after bile duct ligation [24]. Obviously, further experimental work is necessary to determine whether these TLR-dependent processes also apply to primary macrophages and (myo)fibroblasts in the kidney during renal progressive injury. Together, these data suggests that the activation of TLR2 on tubular epithelial and interstitial cells by endogenous stress ligands may play a role in renal inflammation, and apoptosis during progressive renal injury. Indeed, mice deficient for TLR2 had less tubular apoptosis as well as an impaired inflammatory response compared to wild type mice. These data underline the current concept proposing that TLRs play a central role in the pathogenesis of a number of kidney disease [44], [45].

Of particular interest is the finding that TLR2^−/−^ mice had less TEC apoptosis 7 days after UUO-injury while the amount of proliferation was equal as compared to TLR2^+/+^ mice. This could be explained by the reduction in TGF-β and the decrease in the amount of neutrophils we found in these animals. Indeed, both neutrophils and TGF-β are involved in the induction of tubular cell apoptosis in UUO [46]. The lower amount of TGF-β in TLR2^−/−^ mice could reflect the reduced TLR2-dependent synthesis of this factor by TECs, macrophages, and interstitial fibroblasts. The reduction in tubular apoptosis in TLR2^−/−^ mice can also be explained by studies demonstrating a proapoptotic function of TLR2 [47], [48]. In line, we already found that tubular apoptosis was markedly lower in kidneys from TLR2^−/−^ mice than that in kidneys from TLR2^+/+^ mice after renal ischemic injury [6]. We also observed significantly less regeneration of TECs in TLR2^−/−^ mice as compared to TLR2^+/+^ animals after 14 days of UUO. This could reflect the reduced tubular apoptosis in these animals or could imply that TLR2 is necessary for tissue repair [49].

This study presents the first description of the expression, localization and role of TLR2 in renal progressive injury which is characterized by inflammation, apoptosis and fibrosis. We found that TLR2 initiates a renal inflammatory response during obstructive nephropathy but does not play a significant role in the development of renal progressive injury and fibrosis. These results shed new light on the way tissue can monitor chronic cellular injury and subsequently can initiate an inflammatory response.

## Materials and Methods

### Ethics Statement

The Animal Care and Use Committee of the University of Amsterdam approved all animal experiments. Experiments have been conducted according to national guidelines.

### Patients

Four human hydronephrotic obstructed kidneys with extensive fibrosis and tubulus atrophy were removed from patients with end-stage renal disease at the Academic Medical Center. Fifteen renal biopsies of a retrospective study of stored samples were obtained from patients undergoing diagnostic evaluation before therapy was started. The diagnosis IgA nephropathy was based on light microscopic (HE, periodic acid-Schiff, methenamine silver, and alcian blue), and immunofluorescence (IgA, IgG, IgM, C3, C1Q, and light chains) analyses. Patients with coexisting diseases were excluded. We graded renal biopsy specimens according to the classifications of Haas [50] and analyzed biopsies for TLR2 of patients with severe IgA nephropathy (class IV and V). Clinical and biochemical parameters are described before [51]. Five patients with non-IgA nephropathy were included as controls, among which patients with minimal change nephropathy, and thin basement membrane disease that had no pathologic change by light microscopy. All renal biopsies were taken for diagnostic purposes only. This research project used left-over biological material, anonymised and delinked from patient records, and as such was not subject to any requirement for ethical review or approval.

### Mice

Pathogen-free 9- to 12-wk-old male C57BL/6 Wt (TLR2^+/+^) mice were purchased from Charles River. TLR2^−/−^ mice were backcrossed to C57BL/6 background six times, generated as described previously [52], and bred in the animal facility of the Academic Medical Center. Age- and sex-matched mice were used.

### Unilateral Ureter Obstruction

Renal progressive injury was induced as described previously [53], [54]. Briefly, the right ureter was ligated under general anesthesia (0.07 ml/10 g mouse of FFM mixture, containing: 1.25 mg/ml midazolam (Roche), 0.08 mg/ml fentanyl citrate, and 2.5 mg/ml fluanisone (Janssen Pharmaceutica). The abdomen was closed, and mice received a subcutaneous injection of 50 µg/kg buprenorphin (Temgesic, Shering-Plough) for analgetic purposes. Contralateral nonobstructed kidneys served as control. Mice (n = 6 per group) were sacrificed 3, 7, and 14 days after surgery.

### RNA purification and RT-PCR

Total RNA was extracted from renal tissue sections (n = 6 per group) with Trizol reagent (Invitrogen) and converted to cDNA. TLR2, heat-shock protein (HSP)60, HSP70, Gp96, biglycan, high mobility group box chromosomal protein(HMGB)1, MMP2, MMP9, TIMP1, and TIMP2 were analyzed by RT-PCR with SYBR green PCR master mix. Specific gene expression was normalized to mouse hypoxanthine-guanine-phosphoribosyltransferase (HPRT) gene expression. SYBR green dye intensity was analyzed with linear regression analysis. Primer sequences are as follows: HPRT forward primer 5′-tcctcctcagaccgctttt and reverse primer 5′-cctggttcatcatcgctaatc; TLR2 forward primer, 5′- ggggcttcacttctctgctt; reverse primer, 5′-agcatcctctgagatttgacg; Gp96 forward primer, 5′-cggtcaggatatcttctaccagac; reverse primer, 5′-ttcttcctccacctgtgctt; biglycan forward primer, 5′-tcaagctcctccaggttgtc; reverse primer, 5′-gccattatagtaggccctcttg; HMGB1 forward primer, 5′-gagagatgtggaacaacactgc; reverse primer, 5′-ctgtaggcagcaatatccttctc; MMP-2 forward primer, 5′-ataacctggatgccgtcgt; reverse primer, 5′-tcacgctcttgagactttgg; MMP-9 forward primer, 5′-acgacatagacggcatcca; reverse primer, 5′-gctgtggttcagttgtggtg; TIMP-1 forward primer, 5′-tgcacagtgtttccctgttt; reverse primer, 5′-gacggctctggtagtcctca; and TIMP-2 forward primer, 5′-tggacgttggaggaaagaag; reverse primer, 5′-acagagggtaatgtgcatcttg.

### TLR2 expression

Five µm frozen tissue sections were prepared, fixed in ice-cold aceton and endogenous peroxidase activity and non-specific binding were blocked with 0.3% H_2_O_2_ containing sodium azide and 5% normal goat serum (Dako). Sections were stained for TLR2 (anti-mouse/human TLR2, T2.5, eBioscience), incubated with powervision block (human sections, Immunologic), powervision poly-HRP-GAM/R/R IgG (human sections, Immunologic) or goat anti mouse IgG1 HRP (murine sections, Southern Biotech). The TLR2 Ab was proven to be highly specific for both murine and human TLR2 and did not bind to murine TLR2^−/−^ macrophages [25] nor to TLR2^−/−^ granulocytes and monocytes or renal cells of TLR2-antisense-treated mice [6]. Staining without applying a first antibody served as negative control. Slides were developed using DAB (human sections, Sigma) or AEC (murine sections, Sigma). The slides were counterstained with methyl green (human sections, Sigma) or hematoxylin (murine sections).

### Double staining

Frozen sections of human renal biopsies were treated according to the prescribed method for TLR2 histochemistry. Subsequently the specimens were incubated with a cocktail of IgG1Κ anti-TLR2 mAb (T2.5, eBioscience), anti-α-SMA (1A4, msIgG2a, Dako), anti-CD68 (EBM11, Dako) which we labeled with DIG, and anti-CD15 (MMA, NeoMarkers), and incubated with goatα mouse IgG1-HRP (Southern Biotech) or goat α mouse IgG1-AP (Southern Biotech) for TLR2, goat α mouse IgG2a-AP for α-Sma (Southern Biotech), sheep α DIG-AP (Roche) for CD68, or goat α mouse IgM-HRP (Southern Biotech) for CD15 in PBS and 5% normal human serum. Slides were developed using Vector Blue alkaline phosphatase substrate kit III (Vector Laboratories) along with Levamisole (Dako) and Vector NovaRed sustrate kit for peroxidase (Vector Laboratories). For optimal evaluation of double staining, we used spectral imaging and image analysis software (Nuance spectral imaging system, CRi; Woburn) to improve perceptibility of each individual color in immune double stains.

### Gp96, biglycan and HMGB1 expression

Tissue sections of kidneys were deparaffinized and boiled for 10 min in 10 mM sodium citrate buffer (pH 6.0). Non-specific binding and endogenous peroxidase activity were blocked with 0.3% H_2_O_2_ in 100% methanol and 10% normal goat serum (Dako). Sections were then exposed to rat-anti GRP94 (Gp96, Abcam), goat-anti biglycan (Abcam) and rabbit-anti HMGB1 (Abcam), followed by a further incubation with rabbit-anti rat biotine (for GRP94, Dako), swine-anti goat HRP (for biglycan, Biosource), and horseradish peroxidase (HRP)-conjugated goat-anti rabbit IgG (for HMGB1, Immunovision Technology). Gp96 sections were subsequently incubated with avidin-biotin complex (ABComplex/HRP, DAKO, UK). Slides were finally developed using 1% H_2_O_2_ and DAB (Sigma) in 0.05 M Tris-HCl (pH 7.9). The slides were counterstained with methyl green (Sigma). Staining without applying a first antibody served as negative control.

### Histopathological scoring

All histopathological scorings were made in the cortex using PAS-D-stained renal tissue sections and performed by a pathologist on coded slides. The percentage of damaged tubules in the cortex was estimated using a four-point scale according to the following criteria: tubular dilatation, epithelial simplification, and interstitial expansions in ten randomly chosen, non-overlapping fields (×400 magnification). Lesions were graded on a scale from 0 to 4: 0 = normal; 1 = mild, involvement of less than 25% of the cortex; 2 = moderate, involvement of 25% to 50% of the cortex; 3 = severe, involvement of 50 to 75% of the cortex; 4 = extensive damage involving more than 75% of the cortex.

### Detection of collagen, granulocytes, macrophages, and myofibroblasts

Renal tissues were fixed in 10% formalin and embedded in paraffin. Four µm sections were deparaffinized, and digested with a solution of 0.25% pepsine (Sigma) in 0.01 M HCl for granulocyte and collagen type III detection, or boiled for 10 min in 10 mM sodium citrate buffer (pH 6.0) for collagen type I, macrophage, and myofibroblast detection. Subsequently, endogenous peroxidase activity and non-specific binding were blocked as described and sections were respectively exposed to FITC-labeled anti-mouse Ly-6G mAb (Pharmingen), rabbit polyclonal to collagen type I (GeneTex), rabbit polyclonal to collagen type III (Biologo), rat anti-mouse F4/80 IgG2b mAb (Serotec), or mouse-α-human-αSMA-IgG2a (DAKO). For staining of granulocytes, slides were incubated with rabbit anti-FITC antibody (Dako) and peroxidase (HRP)-conjugated goat anti-rabbit IgG (Immunovision Technology). For staining of collagen type I and III slides were incubated with peroxidase (HRP)-conjugated goat anti-rabbit IgG (Immunovision Technology). For macrophage staining, slides were incubated with rabbit anti-rat biotin (Dako), followed by streptavidin-ABC solution (Dako). For staining of myofibroblasts, slides were incubated with goat anti-mouse IgG2a-HRP (Southern Biotech). The slides were developed using 1% H_2_O_2_ and DAB in 0.05 M Tris-HCl (pH 7.9), and counterstained with methyl green. The number of Ly-6G-positive cells were counted in 10 non-overlapping fields (×400). The severity of interstitial fibrosis was additionally evaluated histologically using light microscopy on paraffin-embedded sections stained with picrosirius red that identifies interstitial collagen fibers. The percentage of positive staining for collagen, α-SMA, and F4/80 was analyzed using a computer-assisted digital analysis program (Image Pro-plus®, Mediacybernetyics). At least 15 visual fields were sampled from the cortex and medulla of each kidney using a 20× objective.

### Evaluation of Metalloproteinase (MMP) Activity by Gel Zymography

Frozen tissue sections were sonicated in RIPA buffer (Sigma). Equal quantities of protein (10 µg) were loaded onto a 10% sodium dodecyl sulfate (SDS)-polyacrylamide gel containing 2 mg/ml gelatin (Bloom 225, Sigma) next to a protein marker. To induce MMP activity, gels were incubated overnight at 37°C in a buffer containing 50 mM Tris HCl (pH 7.5), 200 mM NaCl, 5 mM CaCl_2_ and 0.02% Brij-35 (Sigma). To visualize MMP activity, gels were stained with Coomassie Brilliant Blue, destained and subsequently photographed.

### Preparation of renal tissue for cytokine measurements

For cytokine measurements, snap frozen kidneys were diluted in lysis buffer (150 mM NaCl, 15 mM Tris, 1 mM MgCl_2_ pH 7.4, 1 mM CaCl_2_, 1% Triton, 100 µg/ml pepstatin A, leupeptin and aprotinin), homogenized and incubated at 4°C for 30 min. Homogenates were subsequently centrifuged at 1500 g at 4°C for 15 min, and supernatants were stored at −70°C until assays were performed.

### ELISA

Cytokines and chemokines were measured in kidney homogenates using specific ELISAs (R&D Systems) according to the manufacturers' instructions. Activation of latent TGFβ is done according to the protocol supplied by the manufacturer. Renal HGF was measured by ELISA using a mouse anti-HGF antibody (R&D ystems) and a goat anti-HGF (R&D systems) following standard procedures. The detection limits were 12 pg/ml for KC, 7 pg/ml for MCP-1, 125 pg/ml HGF, and 16 pg/ml TGF-β. Cytokine levels were corrected for the amount of protein present using the BioRad protein assay (BioRad Laboratories) with IgG as standard.

### Detection of apoptosis and proliferation

To evaluate the degree of tubular proliferation and apoptosis, tubular Ki67 and active caspase-3 indices were, respectively, determined by counting numbers of positive tubular cells in at least 10 randomly chosen nonoverlapping fields (400 high-power field (hpf)).Tissue sections of kidneys were deparaffinized and boiled for 10 min in 10 mM sodium citrate buffer (pH 6.0). Non-specific binding and endogenous peroxidase activity were blocked as described, followed by an incubation with rabbit anti-human active caspase 3 polyclonal antibody (Cell Signaling), rabbit-anti-Ki67 antibody (DAKO) followed by incubation with peroxidase (HRP)-conjugated goat-anti rabbit IgG (Immunovision Technology), and processed as described above. Slides were finally developed using 1% H_2_O_2_ and DAB (Sigma) in 0.05 M Tris-HCl (pH 7.9) and counterstained with methyl green (Sigma).

### Statistical analysis

Differences between groups were analyzed using Mann-Whitney U test. Values are expressed as mean±standard error of the mean (SEM). A value of p<0.05 was considered statistically significant.
